# Patient satisfaction and its socio-demographic correlates in a tertiary public hospital in Nepal: a cross-sectional study

**DOI:** 10.1186/s12913-021-06155-3

**Published:** 2021-02-12

**Authors:** Mukesh Adhikari, Narendra Raj Paudel, Shiva Raj Mishra, Archana Shrestha, Dipak Prasad Upadhyaya

**Affiliations:** 1 Department of Health Services, Ministry of Health and Population, Kathmandu, Nepal; 2 Department of Health Policy and Management, Yale School of Public Health, Yale University, New Haven, CT USA; 3 Institute for Implementation Science and Health, Kathmandu, Nepal; 4 Public Administration Campus, Tribhuvan University, Kirtipur, Nepal; 5 World Heart Federation Rue de Malatrex 32, 1201 Geneva, Switzerland; 6 Nepal Development Society, Bharatpur-10, Chitwan Nepal; 7 Department of Public Health, Kathmandu University, Kathmandu, Nepal; 8 Central Department of Public Health, Institute of Medicine, Tribhuvan University, Kathmandu, Nepal; 9 School of Medicine, Case Western Reserve University, Cleveland, OH USA

**Keywords:** Patient satisfaction, Outpatient, Public hospital, Sociodemographic, Nepal

## Abstract

**Background:**

Patient satisfaction is one proxy indicator of the health care quality; however, enhancing patient satisfaction in low-income settings is very challenging due to the inadequacy of resources as well as low health literacy among patients. In this study, we assess patient satisfaction and its correlates in a tertiary public hospital in Nepal.

**Methods:**

We conducted a cross sectional study at outpatient department of Bhaktapur Hospital of Nepal. To recruit participants for the study, we applied a systematic random sampling method. Our study used a validated Patient Satisfaction Questionnaire III (PSQ-III) developed by RAND Corporation including various contextual socio-demographic characteristics. We calculated mean score and percentages of satisfaction across seven dimensions of patient satisfaction. To determine the association between various dimensions of patient satisfaction and socio-demographic characteristics of the patient, we used a multi-ordinal logistic regression.

**Results:**

Among 204 patients, we observed a wide variation in patient satisfaction across seven dimensions. About 39% of patients were satisfied in the dimension of general satisfaction, 92% in interpersonal manner, and 45% in accessibility and convenience. Sociodemographic factors such as age (AOR: 6.42; CI: 1.30–35.05), gender (AOR: 2.81; CI: 1.41–5.74), and ethnicity (AOR: 0.26; CI: 0.08–0.77) were associated with general satisfaction of the patients. Other sociodemographic variables such as education, occupation, and religion were associated with a majority of the dimensions of patient satisfaction (*p* < 0.05). Age was found to be the strongest predictor of patient satisfaction in five out of seven dimensions.

**Conclusions:**

We concluded that patient satisfaction varies across different dimensions. Therefore, targeted interventions that direct to improve the dimensions of patient satisfaction where the proportion of satisfaction is low are needed. Similar studies should be conducted regularly at different levels of health facilities across the country to capture a wider picture of patient satisfaction at various levels.

**Supplementary Information:**

The online version contains supplementary material available at 10.1186/s12913-021-06155-3.

## Introduction

Patient satisfaction affects clinical processes and patient outcomes. Various studies have shown that positive patient outcomes are associated with increased patient satisfaction [1, 2]. A prospective study showed that the satisfied patient had two times higher odds of improved quality of life [3]. Evidence from a systematic review showed that patient satisfaction was positively associated with patient safety, clinical effectiveness, compliance to recommended care, and the use of screening services [4]. Similarly, a prior study demonstrated that patients in the highest satisfaction quartile were less likely to visit hospital emergency departments compared to those in the lowest satisfaction quartile [5]. Further, higher patient satisfaction was found to be associated with decreased use of specialty care, hospitalization, and laboratory services [6]. Realizing various positive outcomes, patient satisfaction has been adopted widely in developed countries as an index of health care quality [7, 8].

In contrast to developed countries, the use of patient satisfaction metrics in low-income countries has been limited [9]. Resource constraints in low-income settings have led to a more urgent focus on the availability of basic supplies and services rather than a focus on quality in these settings. Nepal is a low-income country in South Asia that has not highly prioritized improving patient satisfaction. A survey of health facilities in Nepal demonstrated that only 3% of facilities have a functional client feedback system. It further showed that about 23% of the caretakers of sick children were dissatisfied with the services provided by higher-level hospitals due to long waiting times [10].

Patient satisfaction can be affected by a variety of factors, including a patient’s individual characteristics. Association between these characteristics such as age, gender, educational status, religion, race, marital status (etc.), and patient satisfaction are widely inconsistent and contradictory across studies [11]. In addition to individual characteristics, clinical settings—either outpatient or inpatient—have been found to modulate patient satisfaction. Evidence suggests, for example, greater satisfaction in the outpatient reconstruction of anterior cruciate ligament compared to the same surgery in an inpatient setting [12]. These individual characteristics and clinical settings are different in developed countries compared to low-income countries like Nepal and they need to be explored.

Despite Nepal’s renewed commitment to improving patient satisfaction, little progress has been made to understand the level and correlates of patient satisfaction [13]. Indeed, very few studies have been conducted on patient satisfaction, with most of them occurring in specific settings such as maternity care, inpatient settings, eye care, and physiotherapy units. Recently, Poudel et al. conducted a patient satisfaction study in the out-patient department of a public tertiary hospital; however, this study had sample size of less than 100 [14]. Therefore, to address the dearth of literature in patient satisfaction in out-patient settings in Nepal, this study aims to assess patient satisfaction and determine various factors associated with satisfaction in the outpatient settings in a tertiary public hospital.

## Methods

### Study design and settings

We conducted a cross-sectional study in Bhaktapur provincial hospital which has a 75-bed capacity serving approximately 1000 patients in the emergency department and 9000 in the outpatient department every month [15]. The hospital provides a range of services including outpatient, inpatient, emergency, laboratory, and radiology services.

### Sample size and recruitment

We recruited 204 patients from the Out-Patient Department (OPD) of Bhaktapur Hospital from May 7, 2019, to May 22, 2019. We excluded the patients if they were severely ill, were 10 years or younger, or were unable to communicate verbally. The sample size was calculated using 95% confidence interval, 5% margin of error, 86% of patient satisfaction- as observed in a previous study from a similar setting [16], and a 10% non-response rate. Our sampling frame included patients receiving OPD services. We recruited 15 patients per day until the required sample size was achieved. Using systematic random sampling, we recruited every 20th participant every day from the hospital registration, starting with the first OPD patient. If the selected patient refused to participate or was ineligible, we recruited the next patient.

Ethics approval of the study was obtained from the Institutional Review Committee of Kastamandap School of Public Affairs Management, Kathmandu, Nepal. Written consent was obtained prior to the collection of data. For illiterate patients, we read the informed consent form and obtained their verbal consent since the risks associated with our study were low and the potential harm for participants was unlikely.

### Data collection

We collected data through face to face interviews using a patient satisfaction questionnaire (PSQ-III) developed by RAND Corporation [17], the questionnaire is available for free in public domain [18]. Interviews were conducted in the Nepali language. Each interview took about 20–25 min to complete. The questionnaire consists of 18 items probing seven dimensions of patient satisfaction: general satisfaction, technical quality, interpersonal manner, communication, financial aspects, time spent with the doctor, and accessibility and convenience. Each question in the PSQ-III has a 5-point Likert Scale ranging from “Strongly Disagree,” “Disagree,” “Neutral,” “Agree” to “Strongly Agree.” After translating the questionnaire into the Nepali language, we pre-tested in 20 patients at Bhaktapur Hospital. The internal consistency reliability of the items within the seven dimensions was above 0.8 which was above acceptable limit of 0.7 [19]. After pre-testing, we revised the flow of the Nepali language, and added one independent variable, whether the respondent was enrolled in the health insurance program. Realizing the potential role of health insurance in patient satisfaction and the recent initiation of health insurance program in our study hospital, we added this variable in our study.

Further, we also collected sociodemographic information of the participants, including age, gender, ethnicity, religion, marital status, occupational status, educational status, the distance of health facility from their residents, and their enrollment into government health insurance. These data were used as the predictors of the patient satisfaction in the analyses.

### Statistical analyses

Sociodemographic characteristics of the respondents were described in frequency and percentages. We calculated the mean and standard deviation of the Likert scale of each item of PSQ-III. Further, we calculated the frequencies and percentages of satisfied, neutral, and dissatisfied patients.

According to the guidelines of the patient satisfaction questionnaire (PSQ-III) (see Supplemental File Table 1), we classified the satisfaction in each item as follows:

I. ‘Strongly agree’ or ‘Agree’ = Satisfied for Items: 1, 2, 3, 5, 6, 8, 11, 15 and 18
II. ‘Strongly disagree’ or ‘Disagree’ = Satisfied for Items: 4, 7, 9, 10, 12, 13, 14, 16 and 17
III. For all items, the score ranges from 1 (strongly dissatisfied) to 5 (strongly satisfied). The mean score for each item was calculated in the manner that higher the score more the satisfaction level for all the items in the PSQ-III.

To calculate the overall score in each domain, we averaged the score of designated items for each domain as guided by PSQ-III, which is as follows (Supplemental File 1, Table 2).

I. General Satisfaction: Item 3 + 17
II. Technical Quality: Item 2 + 4 +  6 + 14
III. Interpersonal Manner: Item 10 + 11
IV. Communication: Item 1 + 13
V. Financial Aspects: Item 5 + 7
VI. Time Spent with Doctor: Item 12 + 15
VII. Accessibility and Convenience: Item 8 + 9 + 16 + 18

After categorizing the satisfaction score of each domain into three ordinal variables: satisfied, neutral, and dissatisfied, we utilized ordinal logistic regression (OLR) to assess sociodemographic variables associated with satisfaction level. For each patient satisfaction’s domain, we first included all the sociodemographic variables in the model and created the final model that had the lowest Akaike Information Criterion (AIC) through backward selection. We reported adjusted odds ratios with a 95% confidence interval and a *p*-value. A p-value of less than 0.05 was considered significant.

## Findings

### Socio-demographic characteristics

The sociodemographic characteristics of 204 participants are presented in Table 1. The mean age of patients was 39.1 (SD ± 16.6). More than half of the respondents (52.9%) were female. The majority of the patients were married (81.9%). Among the respondents, Hindu was the most common religion (90.2%). About half of the patients (51%) reported that they had to travel less than 30-min walking distance to reach the hospital. The majority of the respondents had formal education (62.7%).

**Table 1:** Sociodemographic characteristics of the respondents

Socio-demographic characteristics	Frequency (%) (***n*** = 204)
**Age**	
Mean (SD)	39.1 (16.6)
**Sex**	
Female	108 (52.9)
Male	96 (47.1)
**Ethnicity**	
Janajati	114 (55.9)
Brahmin/Chhetri	66 (32.4)
Dalit	20 (9.8)
Madhesi	4 (2.0)
**Marital Status**	
Married	167 (81.9)
Unmarried	37 (18.1)
**Religion**	
Hindu	184 (90.2)
Buddhist	14 (6.9)
Others	6 (2.9)
**Distance from health facility**	
Less than 30 min	104 (51.0)
30–60 min	68 (33.3)
More than 1 h	32 (15.7)
**Educational Status**	
Illiterate	32 (15.7)
Informal Education	44 (21.6)
Primary Education	28 (13.7)
Secondary Education	67 (32.8)
Bachelor or higher education	33 (16.2)
**Occupation**	
Service	35 (17.2)
Business	53 (26.0)
Agriculture	22 (10.8)
Homemaker	53 (26.0)
Others	41 (20.1)
**Health Insurance**	
No	117 (57.4)
Yes	87 (42.6)

### Patient satisfaction in seven dimensions

Table 2 summarizes the satisfaction level of the participants by each item of PSQ-18. In the domain of general satisfaction, about 38% of participants reported that the medical care they have been receiving is just about perfect. Regarding the items of technical quality’s domain, the majority (83.82%) reported that their doctors were careful to check everything when treating and examining them. Further, in an interpersonal manner, about 96% of patients responded that their doctor behaved with them in a very friendly and courteous manner. Similarly, in the communication domain, a high proportion of patients (92%) responded that the doctors were good about explaining the reason for medical tests. About 84% of patients disagreed that they had to pay for more of their medical care that they could afford. In the domain of time spent with doctors, the majority of patients (74%) said that their doctors usually spent plenty of time with them. In terms of accessibility and convenience, about 97% agreed that they were able to get medical care whenever they need it.

**Table 2:** Satisfaction of patients segregated by each items of Patient Satisfaction Questionnaire

Item	Question	No (Strongly disagree + Disagree) n (%)	Uncertain n (%)	Yes (Strongly Agree + Agree) n (%)	Mean Score	S. D
1.	Doctors are good about explaining the reason for medical tests.	15 (7.35)	2 (0.98)	187 (91.67)	3.95	0.73
2.	I think my doctors’ office has everything needed to provide complete medical care.	72 (35.29)	40 (19.61)	92 (45.10)	3.14	1.05
3.	The medical care I have been receiving is just about perfect.	46 (22.55)	81 (39.71)	77 (37.75)	3.18	0.89
4.	Sometimes doctors make me wonder if their diagnosis is correct.	153 (75)	19 (9.31)	32 (15.69)	3.91	1.08
5.	I feel confident that I can get the medical care I need without being set back financially	102 (50)	4 (1.96)	98 (48.04)	2.95	1.15
6.	When I go for medical care, they are careful to check everything when treating and examining me.	30 (14.71)	3 (1.47)	171 (83.82)	3.84	0.98
7.	I have to pay for more of my medical care than I can afford.	172 (84.31)	0 (0.00)	32 (15.69)	3.98	1.13
8.	I have easy access to the medical specialists I need.	65 (31.86)	33 (16.18)	106 (51.96)	3.25	1.11
9.	When I get medical care, people have to wait too long for emergency treatment.	22 (10.78)	31 (15.20)	151 (74.02)	1.97	1.04
10.	Doctors act too business like and impersonal towards me	180 (88.24)	13 (6.37)	11 (5.39)	4.12	0.78
11.	My doctors treat me in a very friendly and courteous manner	7 (3.43)	1 (0.49)	196 (96.08)	4.20	0.66
12.	Those who provide my medical care sometimes hurry too much when they treat me.	91 (44.61)	25 (12.25)	88 (43.14)	2.94	1.22
13.	Doctors sometimes ignore what I tell them.	129 (63.24)	12 (5.88)	63 (30.88)	3.28	1.17
14.	I have some doubts about the ability of doctors who treat me.	156 (76.47)	25 (12.25)	23 (11.27)	4.01	1.04
15.	Doctors usually spend plenty of time with me.	45 (22.06)	9 (4.41)	150 (73.53)	3.62	1.07
16.	I find it hard to get an appointment for medical care right away.	43 (21.08)	42 (20.59)	119 (58.33)	2.57	1.03
17.	I am dissatisfied with some things about the medical care I receive.	82 (40.20)	31 (15.20)	91 (44.61)	2.86	1.14
18.	I am able to get medical care whenever I need it	5 (2.45)	2 (0.98)	197 (96.57)	4.2	0.59

According to the guidelines of the patient satisfaction questionnaire (PSQ-III) [see Supplemental File]

(i) ‘Strongly agree’ or ‘Agree’ = Satisfied for Items 1, 2, 3, 5, 6, 8, 11, 15 and 18

(ii) ‘Strongly disagree’ or ‘Disagree’ = Satisfied for Items 4, 7, 9, 10, 12, 13, 14, 16 and 17

(iii) For all items, the score ranges from 1 (strongly dissatisfied) to 5 (strongly satisfied). The mean score for each item was calculated in the manner that higher the score more the satisfaction level for all the items in the PSQ-III

Table 3 presents the mean score and the percentage of satisfaction in seven dimensions of patient satisfaction. The highest level of satisfaction (92% satisfied) was observed in the dimension of the interpersonal manner of patient satisfaction. Whereas, the lowest level of satisfaction (39% satisfied) was observed in the dimension of general satisfaction. More than 70% of the patients were satisfied with the technical quality and communication and nearly two-thirds of the patients were satisfied with the financial aspects of medical care.

**Table 3:** Satisfaction in seven dimensions of the Patient Satisfaction Questionnaire

Satisfaction Domain	Mean of each domain	Mean of Satisfaction scale (Average of mean from component items)	SD of each domain	% Satisfied (Average of percentage of satisfaction of items of each dimensions)
General Satisfaction (Item 3 + 17)	6.04	3.02	1.49	38.9
Technical Quality (Item 2 + 4+ 6 + 14)	14.90	3.73	2.59	70.09
Interpersonal Manner (Item 10 + 11)	8.32	4.16	1.06	92.16
Communication (Item 1 + 13)	7.24	3.62	1.50	77.44
Financial Aspects (Item 5 + 7)	6.93	3.47	1.80	66.17
Time Spent with Doctor (Item 12 + 15)	6.56	3.28	1.87	59.07
Accessibility and Convenience (Item 8 + 9 + 16 + 18)	12	2.99	2.09	45.09

(i) The score for each dimension of the patient satisfaction was calculated on the basis of guideline of PSQ-III (see Supplemental File). The mean of satisfaction each scale ranges from 1(strongly dissatisfied) to 5 (strongly satisfied). The greater the mean, the higher the satisfaction level in each dimension

(ii) Satisfaction = Agree + Strongly agree

### Association between patient satisfaction and sociodemographic characteristics

Table 4 summarizes the associations between dimensions of patient satisfaction and socio-demographic characteristics of the patients.

**Table 4:** Association between independent variables and seven domains of patient satisfaction using multi-ordinal logistic regression

Satisfaction Domain	Explanatory Variables	AOR^**a b**^	95% Confidence Interval	***P***-value
**General Satisfaction**	Age category: 40–60 years	6.42	(1.30–35.05)	0.0258*
	Sex: Female	2.81	(1.41–5.74)	0.0037**
	Ethnic group: Others	0.26	(0.08–0.77)	0.0174
	Occupation: Home makers	0.28	(0.09–0.85)	0.0277*
	Occupation: Others	0.15	(0.04–0.52)	0.0035**
**Technical Quality**	Age category: 40–60 years	16.51	(1.15–323.76)	0.0480*
	Ethnic group: Janajati	2.76	(1.05—7.43)	0.0410*
	Education: Up to Secondary level education	11.28	(2.90–49.56)	0.0007***
	Occupation: Business	0.04	(0.00–0.28)	0.0061**
	Occupation: Agriculture	0.01	(0.00–0.14)	0.0014**
	Occupation: Homemaker	0.03	(0.00–0.31)	0.0086**
**Interpersonal Manner**	Religion: Non-Hindu	0.17	(0.03–1.05)	0.0431*
**Communication**	Age category: 40–60 years	8.33	(1.21–68.90)	0.037*
	Ethnic group: Janajati	2.35	(1.01–5.56)	0.0483*
	Education: Illiterate	0.09	(0.02–0.42)	0.0029**
	Education: Informal Education	0.09	(0.02–0.35)	0.0009***
	Occupation: Home maker	5.37	(1.43–21.33)	0.0143*
	People don’t have insurance	0.33	(0.16–0.69)	0.0037 **
**Financial Aspects**	Distance: 30–60 min	3.15	(1.63–6.23)	0.0007***
	Ethnic group: Others	0.29	(0.10–0.79)	0.0165*
	Religion: Non-Hindu	3.65	(1.25–11.98)	0.0230*
**Time Spent with Doctor**	Age category: 20–40 years	7.34	(1.49–41.64)	0.0176*
	Age category: 40–60 years	10.91	(1.88–72.42)	0.0095**
	Age category: > 60 years	17.09	(2.63–127.05)	0.0037**
	Sex: Female	2.86	(1.40–6.02)	0.0047**
	Ethnic group: Others	0.31	(0.10–0.89)	0.0313*
	Religion: Non-Hindu	11.28	(2.73–62.02)	0.0020**
	Education: Informal Education	0.24	(0.08–0.71)	0.0102*
	Occupation: Business	0.29	(0.10–0.83)	0.0237*
**Accessibility and Convenience**	Age category: 40–60 years	4.64	(1.21–18.92)	0.0278*
	Religion: Non-Hindu	3.08	(1.19–8.37)	0.0224*
	Education: Up to Secondary level	2.83	(1.20–7.00)	0.0197*

*P-value less than 0.05 at 5% level of significance

**p- value less than 0.01 at 5% level of significance

***p- value less than 0.001 at 5% level of significance

^a^Odds ratio obtained after adjusting age, sex, marital status, ethnicity, religion, education, occupation, distance from health facility and insurance status

^b^The reference used in the multi-ordinal logistic regression for various sociodemographic variables are as such: the patients of age less than 20 years for age groups; male patients for gender; Hindu for religion, the women having higher than secondary level education for educational category, service sector for occupation, and Brahmin for ethnicity

#### General satisfaction

In this domain, the odds of patients of age group 40 to 60 years reporting to be satisfied compared to neutral and dissatisfied was 6 times that of patients of 20 years or younger (AOR: 6.42; 95% CI = 1.30–35.05). Further, female patients were more likely to be satisfied than male patients (AOR: 2.81; 95% CI: 1.41–5.74).

#### Technical quality

In this domain, the odds of patients having education up to the secondary level to be satisfied compared to neutral and dissatisfied were 11 times that of patients having educational attainment of more than the secondary level (AOR:11.28; 95% CI: 2.90–49.56). Further, patients who reported that agriculture was their primary occupation were less likely to be satisfied than patients who responded that their main occupation was service (AOR: 0.01; 95% CI: 0.00–0.14).

#### Interpersonal manner

We did not find any associations of sociodemographic variables with the satisfaction level of patients in this domain except religion. Patients who reported as non-Hindu were less likely to be satisfied in an interpersonal manner compared to those who reported as Hindu (AOR: 0.17; 95% CI: 0.03–1.05).

#### Communication

In this domain, the odds of homemaker patients to be satisfied compared to neutral and dissatisfied were 5 times that of patients who were service holders (AOR: 5.37; 95% CI: 1.43–21.33). Patients without health insurance were likely to have less satisfaction than those who had health insurance (AOR: 0.33 95% CI: 0.16–0.69).

#### Financial aspects

In this domain, the odds of patients who had to travel 30 to 60 min of walking distance to be satisfied compared to neutral and dissatisfied were 3 times that of patients who had to travel less than 30 min (AOR: 3.15; 95% CI: 1.63–6.23).

#### Time spent with the doctor

The odds of patients over 60 years of age to be satisfied compared to neutral and dissatisfied were 17 times that of patients who are age 20 or younger (AOR: 17.09; 95% CI: 2.63–127.05). Those who had only informal education were less satisfied compared to the patients who had education more than the secondary level in this dimension (OR: 0.24; 95% CI: 0.08–0.71).

#### Accessibility and convenience

The odds of patients who had education up to the secondary level to be satisfied compared to neutral and dissatisfied were nearly 3 times that of patients who had education more than the secondary level (OR: 2.83; 95% CI: 1.20–7.00).

The only sociodemographic variable, i.e. the marital status did not have any significant association with any of the seven dimensions of patient satisfaction.

## Discussion

Our study assessed patient satisfaction across several dimensions in a tertiary care public hospital in Nepal. About 92% of patients were satisfied with the interpersonal manner of doctors; however, only 39% of patients were satisfied in the dimension of general satisfaction. Several socio-demographic factors were associated with seven dimensions of patient satisfaction. Age was found to be the strongest predictor of patient satisfaction across most of the dimensions.

There was a wide variation in patient satisfaction across the seven dimensions in our study. A recent study conducted in the Bir Hospital, the largest public tertiary hospital in Nepal, did not show such a wide variation across the seven dimensions of patient satisfaction [14]. Such difference between that study and our study might be due to difference in service availability. Bir Hospital provides a wide range of services whereas our study hospital provides comparably a limited range of outpatient, inpatient, diagnostic and emergency services. Further, a study in Australia used a similar patient satisfaction questionnaire to that implemented in our study, but did not find such a wide variation across seven dimensions [20]; the percentage of satisfaction in the financial aspect was the highest (87.4%) while the dimension of accessibility and convenience had the lowest level of satisfaction (72.9%) in their study [20]. Such a wide discrepancy between various dimensions between our study and the Australian study may be due to differences in health care settings, financing mechanisms, and the priority given to patient satisfaction by concerned agencies.

In our study, the general satisfaction of the patients was relatively low (39%). Our low general satisfaction rate was mainly driven by item 17 of PSQ-18, “I am dissatisfied by some things about the medical care I receive.” This dissatisfaction may be due to the absence of some facilities or amenities other than medical services such as availability of drinking water, provision of sanitary toilets etc. A shortage or scarcity of such non-medical amenities may have reduced general satisfaction. Contrast to our study, a recent study by Poudel et al. in a tertiary hospital in Nepal showed a general satisfaction rate of 73% [14]. The hospital where they conducted their study is the largest public hospital in Nepal that has some super-specialized services as well; the patients’ disease status and their severity may be totally different in that hospital compared to our study. Further, a study by Holikatti et al. in India observed a general satisfaction rate of 57%, which is higher than that presented in our study [21]. Similarly, a study conducted in North London reported 71% satisfaction on the general satisfaction scale [22]. Various assessments conducted in the Netherlands demonstrated from 79 to 88% general satisfaction [23, 24] along with the upward shifting of patient satisfaction in Dutch university medical centers [25]. These findings contrast with our study’s finding of general satisfaction which was much lower (39%). Such a high difference in general satisfaction could result from a large gap between expectations and reality among patients who participated in our study. At the same time, the disease status, unavailability of desired amenities or facilities in our study site might have reduced the general satisfaction rate.

Further, in the specific domains of interpersonal manner and communication aspects, patient satisfaction was significantly higher in our study. We statistically tested the mean score in the interpersonal manner and communicable aspects with the score of other dimensions; they were statistically significant. A study by Holikatti et al. found that the satisfaction rate in the interpersonal aspects among patients receiving psychiatric services was 71.4% [21]; their finding was much lower compared to our finding of 90% satisfaction in this domain. The higher level of satisfaction in interpersonal aspects in our study was mainly driven by a friendly and courteous manner of doctors. Such differential satisfaction rates in interpersonal manner may be due to difference in the nature and severity of diseases among studies. The patient satisfaction in these two domains also depend upon the communication skills of the doctors as evidenced by previous studies [26–28].

A study conducted in a private hospital in India demonstrated that approximately 91% of the patients were satisfied with the time spent to the doctor [29], while the proportion of satisfaction of patients in this dimension in our study was 59%. This could be due to doctors spending less time with patients and no much effort put into making a doctor-patient relationship in public hospitals—our study hospital is overstretched with nearly 30 visitation per day per doctor [15]. Regarding affordability, a study by Rizal in the eye services at Nepal Medical College showed that 76.8% of the patients were satisfied [30], while our study showed 66% satisfaction with financial aspects. Eye care services are relatively inexpensive in Nepal compared to general OPD services [31]. Although Nepal’s government implemented a health insurance program in Bhaktapur hospital for more than 2 years with the aim of providing easy access to health care, our study showed only 45% of patients were satisfied in the domain of accessibility and convenience. This low level of satisfaction was largely driven by the unavailability of needed medical specialists, and the problems in obtaining appointments for medical care right away.

In our study, the main predictors for the general satisfaction of the patient were age, gender, ethnic groups, education, and occupation. A systematic review of the determinants of patient satisfaction around the world revealed that age was the most important and consistent predictor of patient satisfaction [32]. Similarly, we found a strong association of age and satisfaction across six dimensions of patient satisfaction with a positive association between satisfaction and age. This association could be explained from different perspectives. First, this could be due to differences in perception of treatment; the older people are more experienced with the care process and the potential weaknesses of health care system [33]. Second, the older people are usually more comfortable with paternalistic type of care rather than patient centered care. Similarly, the elderly people may have a lower than that of younger people [33]. Our study showed that the religion of the patient was an important predictor across four dimensions of patient satisfaction. A secondary analysis of data from a health and retirement study conducted in the United States demonstrated that the patients who believed that religion was a very important part of their life exhibited higher levels of patient satisfaction [34]. Consequently, differences in patient satisfaction among religions, such as between followers of Hinduism and non-followers, would constitute an important area for future exploration.

In our study, female patients were more likely to be satisfied in terms of general satisfaction and time spent with doctor. Contrast to our finding, studies in Malaysia [35] and Nigeria [36] showed male patients were more satisfied compared to their counterparts. In our study, we believe this could be due to a greater number of female service providers in Bhaktapur Hospital. Previous studies showed that female patients were more satisfied with female health care providers [37] and the patients of female physicians were more satisfied than those of male physicians [38].

In our study, Janajati ethnic group were more likely to be satisfied than other groups. This may be due to the reason that most of the doctors at our study hospital belonged to same ethnic group.

In our study, overall, the educated patients were more likely to be satisfied. The patient who had higher level of education may have better understanding about the limitation of public health system. Getting expected or higher level of care might have raised their satisfaction level compared to the patient who had lower level of education. Similar to our finding, a study in psychiatric patient in Qatar showed a higher satisfaction among educated patient [36]. However, a study in Iran [39] revealed the educated patient were less satisfied; this might be due to higher level of expectation in educated people.

Although our study contributed new perspectives in the area of patient satisfaction in Nepal, there are some limitations. First, our findings may not be generalized in the hospitals at the district level or below because of differences in the availability of human resources, diagnostics, and health care services. However, our results could be applicable to the secondary and tertiary level hospitals in Nepal as these hospitals provide similar types of services as rendered in our study hospital. Usually, in Nepal, the patient visit to secondary or tertiary hospitals when their illnesses are not properly addressed at primary or district level hospitals [40]. Similar studies at or below the district level could provide more specific findings for primary level hospitals. Second, there could be a number of correlates of patient satisfaction which we didn’t include in our questionnaire due to resource constraints. For example, severity of the patient’s disease status affects their satisfaction. Due to technical complexities to measure severity of illness, we did not assess patient disease status and severity. Apart from some patient characteristics, we did not collect the supply-side factors such as doctors’ attitude towards their work, their remuneration and incentives, and the opportunity for career growth; these may influence service delivery, eventually affecting patient satisfaction. Even though we did not collect data from the perspectives of health workers, our findings from the patient perspectives also provide some signals about doctors’ availability and their behavior towards patients. Third, due to the cross-sectional nature of the study, we can’t assure that the correlates of the patient satisfaction in our study are indeed causal. However, these associations can be used to initiate some interventions to enhance patient satisfaction. A more robust study design that follows the same patients for a number of visits and controls several explanatory variables can give causal associations. Fourth, the translation of PSQ-18 in Nepalese context may affect the finding of the study; however, during pre-testing we evaluated the internal reliability and consistency, which was above the acceptance level. Fifth, due to the application of PSQ-18 in our study, we use the term ‘patient satisfaction’; however, the statements or items in the PSQ-18 are more related to the evaluations and experience of the patients. Therefore, we recommend the practitioners to look for the results based on the findings of each single-item instead of those based on the mean score of the seven dimensions. Despite such limitations, the findings of this study constitute a useful resource for the Nepalese government to formulate plans and programs to improve patient satisfaction, especially in tertiary care hospitals.

## Conclusions

We concluded that patient satisfaction in a public tertiary level hospital is highly heterogeneous across various dimensions. Patients were least satisfied in the general satisfaction domain and most satisfied in the interpersonal domain. Sociodemographic factors such as age, gender, and ethnicity were associated with general satisfaction, whereas education, occupation, and religion were associated with several other aspects of patient satisfaction defined by PSQ-III. Targeted interventions that direct to improve the dimensions of patient satisfaction where the proportion of satisfaction is low are needed. Similar studies should be conducted regularly at different levels of health facilities across the country to capture a wider picture of patient satisfaction at various levels. Future research design that incorporate the individual combination of a wide range of sociodemographic characteristics would benefit the practitioners and managers to get new insights on the satisfaction from homogenous sub-groups of patients.

## Supplementary Information

**Additional file 1: Supplemental file.** Scoring system of Patient Satisfaction Questionnaire III. **Supplemental file: Table 1.** Scoring Items. **Supplemental file: Table 2.** Creating Scale Score.

## Data Availability

Data will be made available upon reasonable request to the corresponding author by email.

## Abbreviations

AOR: Adjusted Odds Ratio
PSQ: Patient Satisfaction Questionnaire
OPD: Out Patient Department
