# Supplementary material for: Patient satisfaction and its socio-demographic correlates in a tertiary public hospital in Nepal: a cross-sectional study
Source: BMC Health Serv Res. 2021 Feb 12;21:135. doi: 10.1186/s12913-021-06155-3 (PMC7881603; doi:10.1186/s12913-021-06155-3)
Supplement: Supplementary file 1 — Additional file 1: Supplemental file. Scoring system of Patient Satisfaction Questionnaire III. Supplemental file: Table 1. Scoring Items. Supplemental file: Table 2. Creating Scale Score. [file 12913_2021_6155_MOESM1_ESM.docx]

**Supplemental File: Scoring system of Patient Satisfaction Questionnaire III**

Instructions for Scoring the PSQ-18

The PSQ-18 yields separate scores for each of seven different subscales: General Satisfaction (Items 3 and 17); Technical Quality (Items 2, 4, 6, and 14); Interpersonal Manner (Items 10 and 11); Communication (Items 1 and 13); Financial Aspects (Items 5 and 7); Time Spent with Doctor (Items 12 and 15); Accessibility and Convenience (Items 8, 9, 16, and 18).

Some PSQ-18 items are worded so that agreement reflects satisfaction with medical care, whereas other items are worded so that agreement reflects dissatisfaction with medical care. All items should be scored so that high scores reflect satisfaction with medical care (see Table 1). After item scoring, items within the same subscale should be averaged together to create the 7 subscale scores.

We recommend that items left blank by respondents (missing data) be ignored when calculating scale scores. In other words, scale scores represent the average for all items in the scale that were answered.

**Supplemental File:** Table 1

Scoring Items

| **Item Numbers** | **Original Response Value** | **Scored Value** |
| --- | --- | --- |
| 1, 2, 3, 5, 6, 8, 11, 15, 18 | 1 | 5 |
|  | 2 | 4 |
|  | 3 | 3 |
|  | 4 | 2 |
|  | 5 | 1 |
|  |  |  |
| 4, 7, 9, 10, 12, 13, 14, 16, 17 | 1 | 1 |
|  | 2 | 2 |
|  | 3 | 3 |
|  | 4 | 4 |
|  | 5 | 5 |

**Supplemental File:** Table 2

Creating Scale Scores

| **Scale** | **Average These Items** |
| --- | --- |
| General Satisfaction | 3, 17 |
| Technical Quality | 2, 4, 6, 17 |
| Interpersonal Manner | 10, 11 |
| Communication | 1, 13 |
| Financial Aspects | 5, 7 |
| Time spent with Doctor | 12, 15 |
| Accessibility and Convenience | 8, 9, 16, 18 |

Note. Items within each scale are averaged after scoring as shown in Supplemental file Table 1.
